# Carbonic anhydrases in the cell wall and plasma membrane of *Arabidopsis thaliana* are required for optimal plant growth on low CO_2_

**DOI:** 10.3389/fmolb.2024.1267046

**Published:** 2024-02-22

**Authors:** Hiruni N. Weerasooriya, David J. Longstreth, Robert J. DiMario, Viviana C. Rosati, Brittany A. Cassel, James V. Moroney

**Affiliations:** Department of Biological Sciences, Louisiana State University, Baton Rouge, LA, United States

**Keywords:** carbonic anhydrase, photosynthesis, plasma membrane, Arabidopsis, cell wall

## Abstract

**Introduction:** Plants have many genes encoding both alpha and beta type carbonic anhydrases. Arabidopsis has eight alpha type and six beta type carbonic anhydrase genes. Individual carbonic anhydrases are localized to specific compartments within the plant cell. In this study, we investigate the roles of αCA2 and βCA4.1 in the growth of the plant *Arabidopsis thaliana* under different CO_2_ regimes.

**Methods:** Here, we identified the intracellular location of αCA2 and βCA4.1 by linking the coding region of each gene to a fluorescent tag. Tissue expression was determined by investigating GUS expression driven by the αCA2 and βCA4.1 promoters. Finally, the role of these proteins in plant growth and photosynthesis was tested in plants with T-DNA insertions in the αCA2 and βCA4 genes.

**Results:** Fluorescently tagged proteins showed that αCA2 is localized to the cell wall and βCA4.1 to the plasma membrane in plant leaves. Both proteins were expressed in roots and shoots. Plants missing either αCA2 or βCA4 did not show any growth defects under the conditions tested in this study. However, if both αCA2 and βCA4 were disrupted, plants had a significantly smaller above- ground fresh weight and rosette area than Wild Type (WT) plants when grown at 200 μL L^−1^ CO_2_ but not at 400 and 1,000 μL L^−1^ CO_2_. Growth of the double mutant plants at 200 μL L^−1^ CO_2_ was restoredif either αCA2 or βCA4.1 was transformed back into the double mutant plants.

**Discussion:** Both the cell wall and plasma membrane CAs, αCA2 and βCA4.1 had to be knocked down to produce an effect on Arabidopsis growth and only when grown in a CO_2_ concentration that was significantly below ambient. This indicates that αCA2 and βCA4.1 have overlapping functions since the growth of lines where only one of these CAs was knocked down was indistinguishable from WT growth. The growth results and cellular locations of the two CAs suggest that together, αCA2 and βCA4.1 play an important role in the delivery of CO_2_ and HCO_3_^−^ to the plant cell.

## 1 Introduction

Carbonic anhydrases (CAs) are ubiquitous in nature, catalyzing the interconversion of CO_2_ and HCO_3_^−^. While most CAs are zinc metalloenzymes, there are multiple structurally and sequentially distinct families found across many different species. Eight different CA families (alpha through iota) have been described to date, with some of the CAs having structural as well as catalytic functions (Hewett-Emmett and Tashian, 1996; Peña et al., 2010; DiMario et al., 2017; Jensen et al., 2020). In addition to catalyzing the CO_2_ and HCO_3_^−^ interconversion, CAs are important in facilitating the movement of CO_2_ and HCO_3_^−^ across membranes. In animals, CAs are important in transferring inorganic carbon out of respiring cells, red blood cells, lungs and kidneys (Occhipinti and Boron, 2019). In plants and algae, CAs are important in the delivery of CO_2_ for photosynthesis. For example, most algae have CO_2_ concentrating mechanisms (CCMs) and CAs play important roles in this process (Mukherjee et al., 2019).

In the unicellular green alga, *Chlamydomonas reinhardtii* (hereafter referred to as Chlamydomonas), there are two αCA isoforms that contribute to the functioning of the alga’s CCM. The first algal αCA, CAH1, is a periplasmic CA whose gene expression is highly upregulated when Chlamydomonas is introduced to a low CO_2_ environment (Fujiwara et al., 1990; Fukuzawa et al., 1990). CAH1 is thought to help facilitate CO_2_ + HCO_3_^−^ movement into the cell from the periplasmic space (Moroney and Ynalvez, 2007). The evidence to support this comes from using the CA inhibitor, acetazolamide, during photosynthesis measurements of Chlamydomonas cultures under various pH conditions (Moroney and Tolbert, 1985). Under high pH conditions, where the predominant inorganic carbon form is HCO_3_^−^, the photosynthesis rate of Chlamydomonas is decreased when acetazolamide inhibits CAH1 whereas the effect of acetazolamide on photosynthesis is much less under acidic conditions where CO_2_ is the predominant inorganic carbon molecule and can freely diffuse into the cell (Moroney and Tolbert, 1985). The other algal αCA, CAH3, is a chloroplast thylakoid lumen CA that relocates to the pyrenoid of Chlamydomonas when it is phosphorylated (Moroney and Ynalvez, 2007; Blanco-Rivero et al., 2012). CAH3 is thought to dehydrate HCO_3_^−^ in the acidic thylakoid lumen to CO_2_ for Rubisco to fix to RuBP. Chlamydomonas mutants lacking CAH3 grow very poorly in a low CO_2_ environment, although they grow normally in the presence of high CO_2_ (Karlsson et al., 1998; Duanmu et al., 2009).

Terrestrial plants have a large number of genes encoding carbonic anhydrases. Arabidopsis is typical with eight α-type and six β-type CAs (DiMario et al., 2017; Langella et al., 2022). The β-type CAs have been localized to the chloroplast, cytoplasm, plasma membrane, and mitochondria (DiMario et al., 2017). α-type CAs are much less studied although a proteomic study by Chen et al. (2009) has shown the presence of an α-type CA in *Oryza sativa* calli. In terrestrial plants, the role(s) of CA in photosynthesis varies with the type of photosynthesis performed by the plant. Plants that use C4-type photosynthesis have a CCM and CAs play an essential role. In contrast, C3 plants rely on the diffusion of CO_2_ into the leaves and do not have an active CCM. In C4 plants such as maize or sugar cane, phosphoenolpyruvate carboxylase (PEPCase) catalyzes the first carboxylation reaction. Since PEPCase uses HCO_3_^−^ as its substrate, CO_2_ entering the mesophyll cell must first be converted to HCO_3_^−^. Maize plants missing the genes encoding the cytoplasmic CAs can no longer concentrate CO_2_ and grow poorly in ambient air (DiMario et al., 2021). The role(s) of CA in C3 photosynthesis is much less clear. Knocking down or knocking out the expression of the predominant chloroplast CA does not reduce photosynthesis (Hines et al., 2021). Recent work knocking out both chloroplast CAs, βCA1 and βCA5, did cause reduced growth but the growth inhibition appeared to be the result of a decrease in lipid biosynthesis (Hines et al., 2021; Weerasooriya et al., 2022). It has been reported that knocking out both chloroplast CAs in tobacco did not affect photosynthesis (Hines et al., 2021). So, while the roles of the CAs in photosynthesis are different in C3- and C4-plants, both have a large number of genes encoding both α- and β-type CAs.

The expression level of CAs in plants is substantial, as it is estimated that CAs account for 1%–2% of the soluble proteins in leaf tissue (Tobin, 1970; Okabe et al., 1984; Peltier et al., 2006). This is true whether the plant performs C3- or C4-type photosynthesis. In *Arabidopsis thaliana* (hereafter referred to as Arabidopsis), a C3 plant, there are a total of eight αCAs and six βCAs found in its genome. In leaves, all six βCAs are expressed and they account for the majority of the CAs found in leaf tissue. RT-PCR,microarray, and RNAseq data show that only αCA1, αCA2, and αCA3 are appreciably expressed in leaves (Fabre et al., 2007; DiMario et al., 2016; Zhang et al., 2020). However, even though many CAs are expressed in leaves, the physiological role of a number of these CAs remains obscure.

CAs are localized to a variety of locations within the plant cell. The location of the six βCAs is well established. The βCA1 and βCA5 proteins are localized to the chloroplast stroma (Fabre et al., 2007; DiMario et al., 2017) while βCA2 and βCA3 are cytoplasmic. βCA4 is made as two isoforms with the longer form (βCA4.1) going to the plasma membrane and the shorter form (βCA4.2) to the cytoplasm.

βCA6 is mitochondrial. The location of the leaf αCAs is far less certain. According to subcellular localization prediction software, αCA1, αCA2, and αCA3 are all predicted to be directed to the endoplasmic reticulum (ER)/secretory pathway (SP). However, little experimental work has been conducted on the final subcellular localization of these proteins. Alpha CA1 has been reported to be in the chloroplast but no role for this protein has been discovered at this time (Villarejo et al., 2005). Despite the study of the novel subcellular localization pathway of αCA1, no subcellular localization experiments have been conducted on αCA2 and αCA3. There has yet to be any published work on transgenic plants expressing αCA1, αCA2, and αCA3 GFP constructs in Arabidopsis.

Therefore, the first goal of this work was to establish the localization of the αCA2 protein in Arabidopsis. The second goal was to study the physiological role of αCA2 and βCA4.1 proteins. Our aim was to determine whether these CAs help deliver CO_2_ to supply the plant with CO_2_ and HCO_3_^−^. To answer this question, we studied the phenotypes of *αCA2βCA4* double mutants, grown under different CO_2_ conditions. The purpose of this communication is to show the importance of cell wall and plasma membrane CAs for normal plant growth under low CO_2_ conditions.

## 2 Materials and methods

### 2.1 Plant lines and growth conditions

All Arabidopsis (*A. thaliana*) Wild Type (WT) and the plants containing T-DNA insertions used in this work are of the Columbia (COL) ecotype. These plants were grown under ambient CO_2_ (400 μL CO_2_ per L air or 400 μL L^−1^), low CO_2_ (200 μL L^−1^), and high CO_2_ (1,000 μL L^−1^) with an 8-h light, 16-h dark cycle with a light intensity of 120 μmol photons m^−2^ sec^−1^. All plants used in growth studies were watered every other day, alternating between distilled H_2_O (dH_2_O) and a 1:3 dilution of Hoagland’s nutrient solution in distilled H_2_O (Epstein and Bloom, 2005).

### 2.2 Construction of vectors for GUS and eGFP expression

Amplicons for pENTR™ Gateway construction were generated using Phusion polymerase (New England Biolabs). Primers for amplifying the coding regions and promoter regions of *αCA2* (At2g28210) and *βCA4* (At1g70410) were designed using Integrated DNA Technologies primer design tools and were generated by Integrated DNA Technologies (see Supplementary Table S1 for primer sequences). PCR fragments were gel purified using the Qiaquick Gel Extraction kit (Qiagen). 1–2 μL of purified PCR product was added to a pENTR™ master mix (1 μL of a 1.2 M NaCl and 0.06 M MgCl_2_ mix [Invitrogen], 1 μL pENTR™/dTOPO^®^ vector mix [Invitrogen], and dH_2_O [Invitrogen] to a final volume of 6 μL) for pENTR™ vector construction. Vectors were transformed into *E. coli* TOP10 chemically competent cells and plated onto YEP plates (for 1 L: 10 g peptone, 5 g NaCl, 10 g yeast extract, 15 g agar) supplemented with 50 μg mL^−1^ kanamycin. pENTR™ vectors were subjected to restriction digestion and sequencing to confirm the correct orientation and sequence of the construct. eGFP amplicons were recombined into the pDEST vector pB7FWG2 (Karimi et al., 2002) and GUS amplicons were recombined into the pDEST vector pKGWFS7 (Karimi et al., 2002). The correct orientation of the pDEST™ vector was confirmed via restriction digestion.

Constructs used for the complementation studies were assembled using the GoldenGate modular cloning system (Weber et al., 2011; Patron et al., 2015). Linear Level 0 gene fragments (promoters, coding regions, terminators) were synthesized by Twist Biosciences (San Francisco, CA, United States) with defined Golden Gate compatible overhangs and cloned via traditional digestion andligation into Golden Gate compatible acceptor plasmids. Next, Level 1 constructs were generated to express the αCA2 or βCA4.1 genes under the control of the desired promoters. Finally, Level 1 constructs were assembled into Level 2 constructs to include a constitutively expressed BASTA resistance cassette. Correct assembly of Level 2 backbones was confirmed via restriction digestion.

### 2.3 *Agrobacterium tumefaciens* transfection and screening of transformants

Stable eGFP, GUS, and complementation lines were created following a modified procedure (Weigel and Glazebrook, 2002). A total of 200 μL of transformed *A. tumefaciens* was used to inoculate 200 mL of LB medium supplemented with antibiotics (30 μg mL^−1^ gentamycin and 10 μg mL^−1^ rifampicin for *A. tumefaciens* helper plasmids and either 100 μg mL^−1^ spectinomycin for the eGFP and GUS vectors or 50 μg mL^−1^ kanamycin for the complementation vector). The cultures were grown overnight at 28°C with vigorous shaking, and cells were pelleted in the morning by centrifugation at 7,250 RCF for 10mins at 20°C using a Beckman J2-HS centrifuge and JA-10 rotor. Pelleted cells were resuspended in 400 mL of *A. tumefaciens* infiltration medium (one-half-strength Murashige and Skoog medium with Gamborg’s vitamins from Caisson Laboratories, 5% [w/v] Sucrose, 0.044 μM benzylaminopurine suspended in dimethyl sulfoxide, and 50 μL L^−1^ Silwet L-77 from Lehle Seeds). Inflorescences of Arabidopsis plants were dipped in the *A. tumefaciens* infiltration medium for approximately 40 s and then laid sideways in a flat tray with a covered dome to recover overnight, before incubating in constant light at 21°C (Weigel and Glazebrook, 2002). Positive transformants were selected on soil by spraying seedlings with a 1:1,000 dilution of BASTA (AgrEvo).

### 2.4 Transient transformation of tobacco leaves for αCA2-mTurquoise expression

Four-to five-week-old *Nicotiana tabacum* (tobacco) plant leaves were used for transient eGFP expression. Two *A. tumefaciens* strains were used to generate transient eGFP expression in tobacco leaves. When infiltrating tobacco leaves, one strain, GV3101 containing the αCA2-mTurquoise construct, was combined with a second *A tumefaciens* strain, AGL-1 [p19], containing a suppressor to gene silencing construct (Voinnet et al., 2003). Two days before infiltrating tobacco leaves with an *A. tumefaciens* solution, 2 mL of YEP liquid culture was inoculated with either a single *A. tumefaciens* colony from an agar plate or from a glycerol stock and placed in a 28°C shaker overnight. The medium which was used to grow GV3101 contained 100 μg mL^−1^ Spectinomycin (MP Biomedicals, LLC), 30 μg mL^−1^ Gentamycin (GOLDBIO), and 10 μg mL^−1^ Rifampicin (GOLDBIO). The medium which was used to grow AGL-1 [p19] also contained 50 μg mL^−1^ Kanamycin (Sigma). The following afternoon, 100 μL of the GV3101 and AGL-1 [p19] cultures were transferred to 5 mL of YEP solution containing the appropriate antibiotics and were placed in a 28°C shaker overnight. Once an OD600 of 2.0 was reached,0.5 mL of the GV3101 culture and 0.5 mL of the AGL-1 [p19] culture were combined in a 2 mL microcentrifuge tube. The sample was spun at 3,625 RCF for 10 min and the supernatant was discarded. The *A. tumefaciens* pellet was resuspended in 1 mL of 10 mM MgCl_2_ to remove the antibiotics. The *A. tumefaciens* solution was spun again at 3625 RCF for 10 min and the supernatant was discarded. The *A. tumefaciens* pellet was resuspended in 2 mL of an *A. tumefaciens* resuspension solution (1 mL of 100 mM MES; 1 mL of 100 mM MgCl_2_; 100 μL of 1.5 Acetosyringone (Sigma); 7.9 mL dH_2_O). Using a needle-less syringe, the 2 mL of *A. tumefaciens* solution was infiltrated into the abaxial side of multiple tobacco leaves. Infiltrated tobacco plants were returned to normal growth conditions and were imaged by confocal microscopy 3 days later.

### 2.5 Histochemical GUS staining

GUS staining was visualized following a modified protocol of Jefferson et al. (1987). Plants and inflorescences were submerged in a GUS staining solution (0.1M NaPO_4_ pH 7, 10 mM EDTA, 0.1% [v/v] Triton X-100, 1 mM K_3_Fe(CN)_6_, 2 mM 5-bromo, 4-chloro, 3-indolβ-D-glucuronic acid [X-Gluc, from GoldBio] suspended in N, N-dimethylformamide) and were placed in a 37°C incubator in the dark overnight. The following morning, plants were taken out of the incubator and the GUS staining solution was aspirated. Plant tissues were incubated in 100% methanol at 60°C for 15 min repeatedly until all chlorophyll was removed.

### 2.6 Protoplast preparation and eGFP and m-Turquoise visualization

Following the protocol of Wu et al. (2009), 2 g of leaf tissue was incubated in 10 mL of enzyme solution (1% [w/v] cellulase from *Trichoderma viride* [Sigma], 0.25% [w/v] pectinase from *Rhizopus* spp. [Sigma], 0.4 M mannitol, 10 mM CaCl_2_, 20 mM KCl, 0.1% [w/v] bovine serum albumin, and 20 mM MES at pH 5.7) for 1 h in light after placing Time Tape on the upper epidermis of the leaves and removing the lower epidermis of the leaves via Magic Tape. Protoplasts were then pelleted by centrifugation at 73 RCF for 3 min at 4°C using a Beckman J2-HS centrifuge and JS-13.1 rotor. Protoplasts were resuspended in a solution containing 0.4 M mannitol, 15 mM MgCl_2_, and 4 mM MES at pH 5.7. eGFP fluorescence was visualized using protoplasts and leaves from stable eGFP plants with a Leica SP2 confocal microscope. The white light laser was used with 5% laser power and smart gain was adjusted to 100%. A 40X oil-emersion lens was used to visualize protoplasts and a ×20 objective lens was used to visualize intact cells from leaf samples. eGFP and chlorophyll were excited using a krypton/argon laser tuned to 488 nm, and eGFP and chlorophyll fluorescence were observed between the wavelengths of 500–520 nm and 660–700 nm, respectively.

### 2.7 Genotyping T-DNA lines using genomic PCR and reverse transcription-PCR

DNA for genomic PCR was isolated from Arabidopsis leaves ground with a mortar and pestle and incubated in Edward’s extraction buffer (200 mM Tris-Cl, pH 7.5, 250 mM NaCl, 25 mM EDTA, and 0.5% [w/v] SDS). DNA was precipitated using 100% isopropanol followed by 70% [v/v] ethanol washes. RNA for reverse transcription was isolated from 80 mg of leaf tissue from 6-week-old Arabidopsis plants grown under ambient CO_2_ and short days using the Qiagen RNeasy Plant minikit. Three micrograms of RNA were used for the reverse transcription reaction, and cDNA was generated using the SuperScript First-Strand RT-PCR kit and protocol (Invitrogen). cDNA at 0.5 μL was used for a 25-μL PCR using the standard protocol for One Taq (New England Biolabs).

### 2.8 Rosette area and fresh weight measurements

Individual plants from each line were photographed weekly, and rosette areas were measured by tracing the outlines of the plants and obtaining the projected rosette area within each outline in ImageJ (National Institutes of Health). Rosette areas were measured on three plants per line. Fresh weights of above-ground plant mass were measured every week for each plant line for 5 weeks. Three plants per line were used for fresh weight analysis.

### 2.9 Gas exchange analysis

Leaf gas-exchange rates were measured with a LI-COR 6800 gas analyzer system and the 6800-fluorometer chamber. Photosynthetic response was characterized by construction of net assimilation rate *versus* leaf intercellular CO_2_ concentration (A/Ci) curves for the 16th youngest leaf from four, separate, 7-week-old plants from each genotype studied. These measurements were made on plants grown at 200, 400 and 1,000 µL L^–1^ CO_2_. Leaves were first allowed to acclimate in the leaf cuvette at 400 μL L^–1^ CO_2_, 1,000 μmol photons m^–2^s^–1^ (saturating irradiance for these leaves), and 23°C–25°C until a steady-state for A was reached. Steady-state A was then measured at 400, 300, 250, 200, 100, 50, 400, 500, 600, 700, 800, 1,000, 1,300, 1,600 and 1,800 μL L^–1^ CO_2_ in air. We statistically analyzed the effects of genotype and growth CO_2_ concentration on three characteristics of the A/Ci curves. The first characteristic was the CO_2_ saturated A (A_csat_) which occurred at Ci values of 1,300–1,600 μL L^–1^ CO_2_. The slope of the initial, linear portion of the A/Ci curve was the second characteristic. The CO_2_ compensation point was the third characteristic. The initial linear portion for each A/Ci curve was determined from a linear regression fitted to the first 3-4 points on the curve (where *r* ^2^ for the regression was 0.95–0.99). The compensation point was the Ci calculated from each regression by setting A equal zero.

### 2.10 Stomatal analysis

Average abaxial and adaxial stomatal density values were taken from 20 WT, *αca2, βca4,* and *αca2βca4* Arabidopsis leaf peels. Student’s t-test was performed to analyze the significant difference in the values among the four genotypes.

### 2.11 Statistical analysis

Statistical analysis for the growth data (Figure 8) was performed using One-way ANOVA in GraphPad Prism 8. Statistical significance is defined as *p* < 0.05. Two-factor ANOVA was performed for the CO_2_ saturated A (Table 1), The slope of the initial, linear portion of the A/Ci curve (Table 2), and the CO_2_ compensation point (Table 3). Statistical significance is defined as *p* < 0.01. Stomatal density (Supplementary Table S2) and stomatal conductance (Supplementary Table S3) values were analyzed using Student’s t-test. Statistical significance is defined as *p* < 0.05. At least three replicates of WT, *αca2*, *βca4*, and *αca2βca4* plants were used for all the analysis.

**TABLE 1:** Mean CO_2_ saturated A (A_csat_) taken from A/Ci curves for the four genotypes grown at three different CO_2_ concentrations. Values are means of the 16th leaf from four different plants for each genotype ± one standard deviation. A two-factor ANOVA (growth CO_2_ concentration x genotype) was highly significant (*p* < 0.01) for both growth CO_2_ concentration and genotype.

Growth CO_2_	Genotype			
	WT	*αca2*	*βca4*	*αca2βca4*
200 μL L^–1^	17.2 ± 2.8	17.5 ± 0.7	16.7 ± 0.9	10.1 ± 1.5
400 μL L^–1^	21.5 ± 1.1	21.5 ± 1.4	20.1 ± 0.5	20.8 ± 1.3
1,000 μL L^–1^	24.2 ± 2.8	25.3 ± 1.2	23.5 ± 4.8	22.0 ± 0.8

**TABLE 2:** Mean initial slopes calculated from the A/Ci curves for the four genotypes grown at three different CO_2_ concentrations. Slopes were from linear regressions fitted to the first 3-4 points on each A/Ci curve. Values are means of the 16th leaf from four different plants for each genotype ± one standard deviation. A two-factor ANOVA (growth CO_2_ concentration x genotype) was highly significant (*p* < 0.01) for both growth CO_2_ concentration and genotype.

Growth CO_2_	Genotype			
	WT	*αca2*	*βca4*	*αca2βca4*
200 μL L^–1^	0.047 ± 0.013	0.047 ± 0.011	0.042 ± 0.002	0.028 ± 0.011
400 μL L^–1^	0.055 ± 0.007	0.059 ± 0.006	0.049 ± 0.003	0.044 ± 0.007
1,000 μL L^–1^	0.057 ± 0.005	0.058 ± 0.015	0.051 ± 0.014	0.049 ± 0.002

**TABLE 3:** Mean CO_2_ compensation points calculated from the A/Ci curves for the four genotypes grown at three different CO_2_ concentrations. Each compensation point was calculated from linear regressions from each A/Ci curve as the value for Ci when A = zero. Values are means from the regression for the 16th leaf from four different plants for each genotype ± one standard deviation. A two-factor ANOVA (genotype x growth CO_2_ concentration) was highly significant (*p* < 0.01) for growth CO_2_ concentration but insignificant for genotype.

Growth CO_2_	Genotype			
	WT	*αca2*	*βca4*	*αca2βca4*
200 μL L^–1^	75.9 ± 13.1	81.1 ± 15.5	80.7 ± 7.9	98.9 ± 14.2
400 μL L^–1^	67.1 ± 1.1	73.5 ± 5.8	66.4 ± 5.3	63.9 ± 7.0
1,000 μL L^–1^	65.8 ± 5.9	71.5 ± 8.7	65.4 ± 7.0	60.9 ± 7.1

## 3 Results

### 3.1 In leaf tissue αCA2 is located in the cell wall and βCA4.1 is on the plasma membrane

**FIGURE 1:**
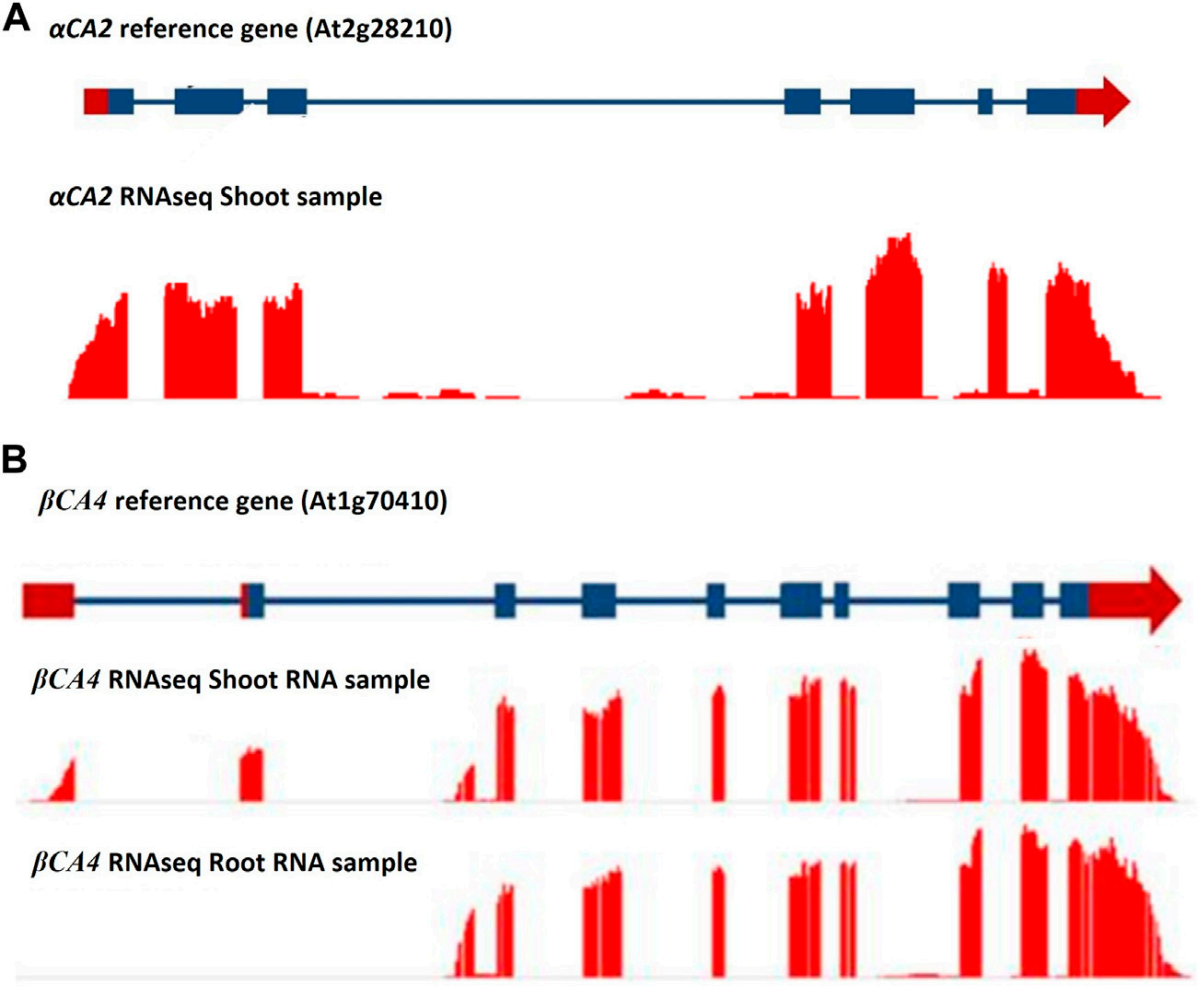
RNAseq data for the *αCA2* and *βCA4* genes. **(A)** RNAseq data for *αCA2* gene show there are seven exons and six introns present in the gene. **(B)** *βCA4* has two mRNA forms. Leaf and root RNA samples yielded two forms of *βCA4* mRNA. The long mRNA form is found primarily in the leaf and contains 10 exons, where the first two exons are unique to the long form. The short mRNA form has nine exons, where the first exon is unique to the short mRNA form and can be found in both theroot and shoot RNA samples. Blue boxes and blue lines represent exons and introns, respectively. Red boxes and red arrows represent the 5′ and 3′ UTRs, respectively.

The αCA2 gene (At2G28210) is composed of five exons with a relatively large intron between exons two and three (Figure 1A). The gene encoding βCA4 (At1g70410) is more complex as it has two active transcription start sites (Figure 1B). The longer transcript, βCA4.1, has ten exons while the shorter form, βCA4.2, is composed of nine exons. Both βCA4.1 and βCA4.2 are present in leaf tissue but only βCA4.2 is found in root tissue (DiMario et al., 2016). Previous work has shown that the shorter version of βCA4.2 is cytoplasmic while βCA4.1 is bound to the plasma membrane (DiMario et al., 2016).

**FIGURE 2:**
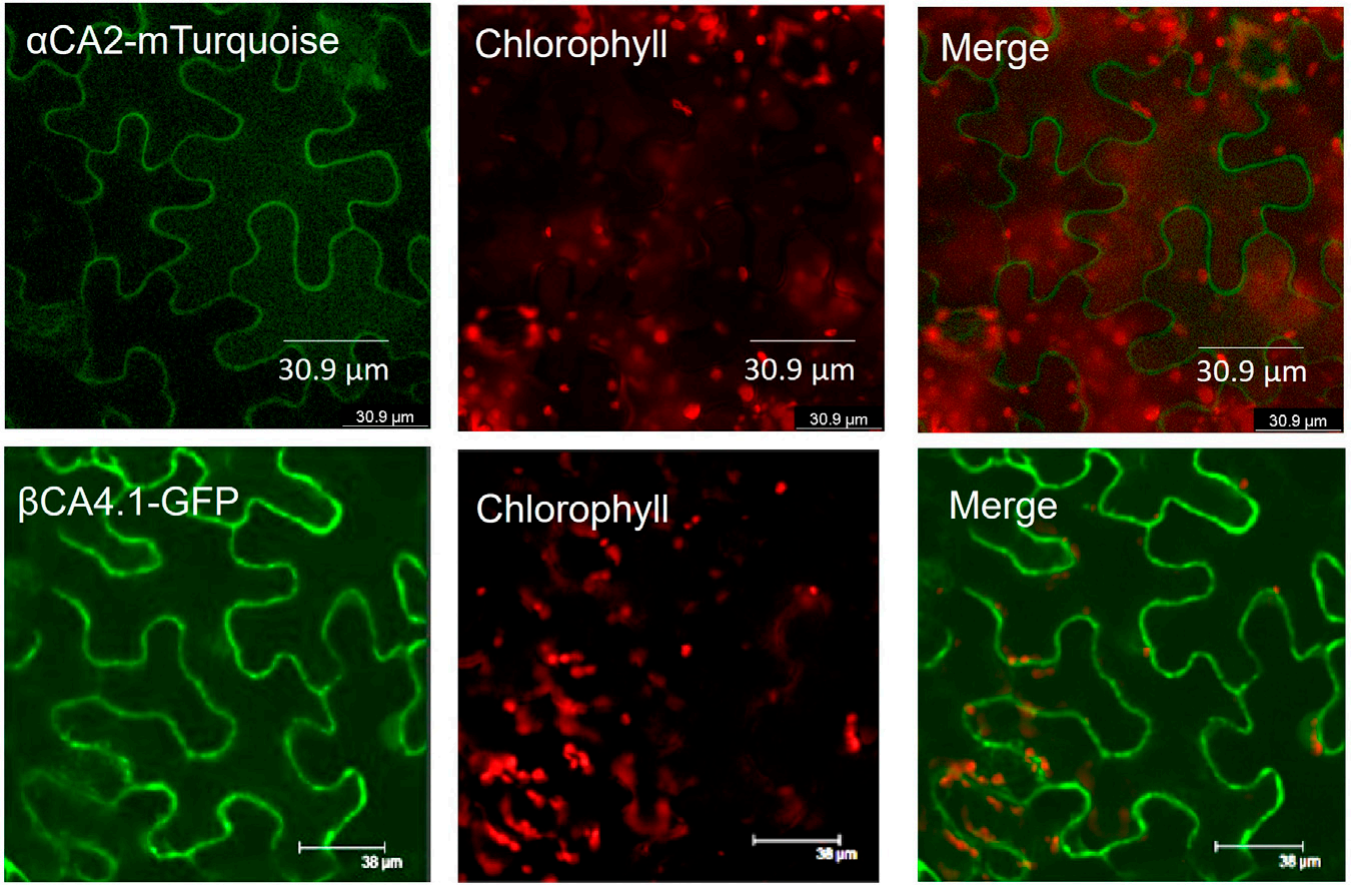
Localization of αCA2 and βCA4.1 proteins. Leaves from transiently expressing αCA2-mTurquoise and stably expressing βCA4.1-eGFP were imaged using confocal microscopy. Green represents eGFP and m-Turquoise fluorescence and red represents chlorophyll autofluorescence.

**FIGURE 3:**
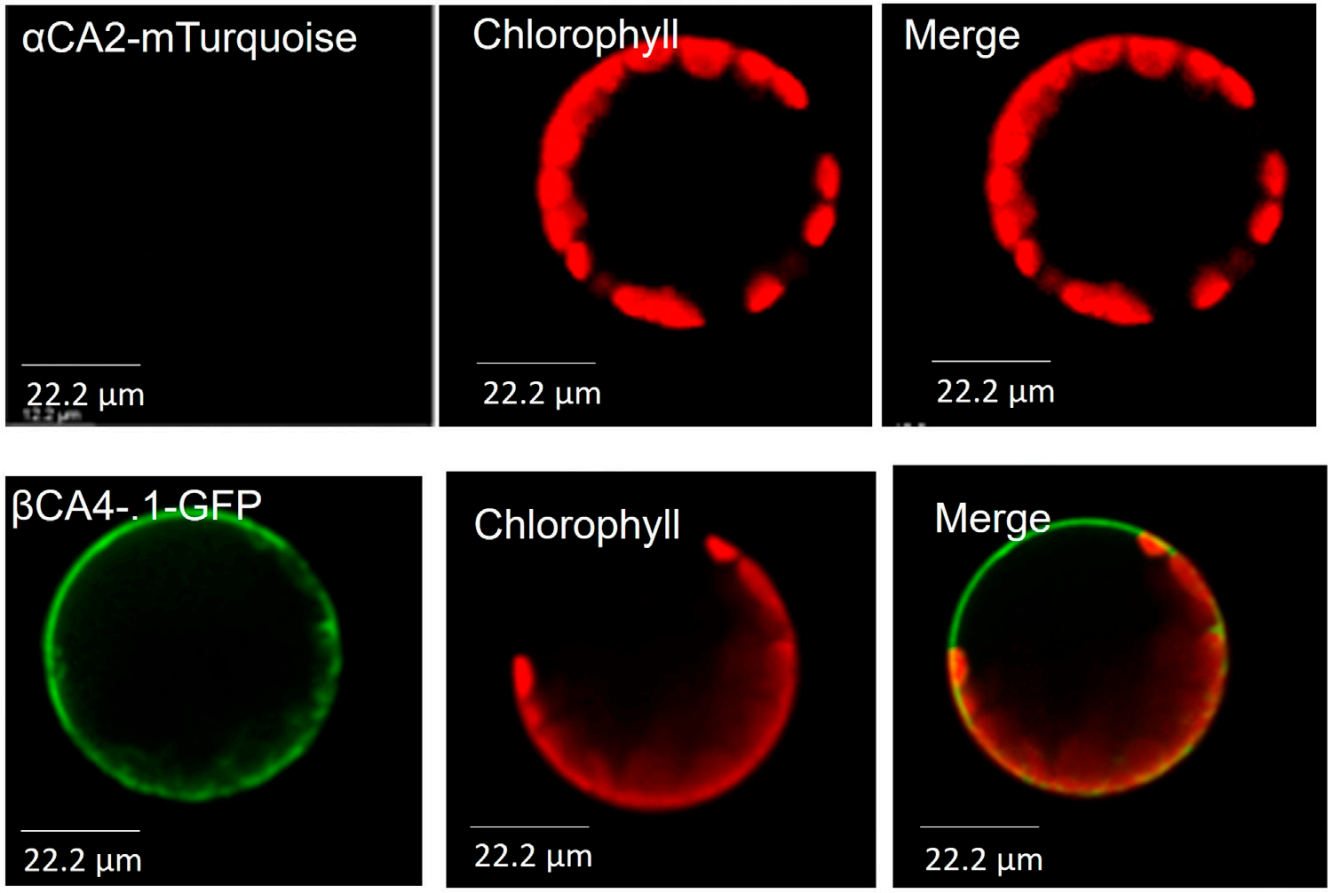
αCA2 and βCA4.1 are cell wall and plasma membrane proteins respectively. Protoplasts from transiently expressing αCA2-mTurquoise and stably expressing βCA4.1-eGFP were imaged using confocal microscopy. Green represents eGFP and m-Turquoise fluorescence and red represents chlorophyll autofluorescence.

To determine the cellular location of αCA2, *αCA2* was initially fused with *GFP*, but this resulted in faint fluorescent signals around the periphery of the leaf cells. To better determine the localization of αCA2 in plant cells, the coding region of *αCA2* was cloned upstream of a C-terminal m-Turquoise tag to generate the *35S::αCA2-m*-*Turquoise* construct. We switched to m-Turquoise as it gives a better fluorescent signature under acidic conditions. Transiently expressed tobacco leaves with *35S::αCA2m-Turquoise* showed the fluorescence signal on the outline of the leaf cells as seen by the clear “jigsaw” pattern in Figure 2. The clear fluorescent signals from m-Turquoise were similar to those seen when a C-terminal eGFP tag was added to *βCA4.1* (Figure 2). When protoplasts were made from leaf cells expressing *βCA4.1-GFP*, the GFP signal was clearly seen around the plasma membrane (Figure 3) confirming the earlier reports by Fabre et al. (2007), Hu et al. (2010), and Hu et al. (2015) that βCA4.1 is located on the plasma membrane. The results shown in Figure 3 are consistent with the RNAseq data (Figure 1) that βCA4.1 is expressed in leaves and it is located on the plasma membrane.

αCA2 is predicted to move through the secretory pathway of Arabidopsis (TargetP 2.0) and the fluorescence pattern seen in plants expressing *35S::αCA2-m-Turquoise* is consistent with the protein going through the secretory pathway to the plasma membrane or cell wall. However, while protoplasts generated from stably transformed Arabidopsis plants expressing *βCA4.1-eGFP* gave a plasma membrane signal (Figure 3), the m-Turquoise signal attached to αCA2 was missing in the transfected protoplasts confirming the cell wall localization (Figure 3). We hypothesize that the m-Turquoise linked to αCA2 is released from the cell as the cell wall is digested during protoplast generation. These results are consistent with αCA2 being a cell wall protein and agree with the software location predictions.

### 3.2 Tissue expression patterns of *αCA2* and *βCA4.1*

**FIGURE 4:**
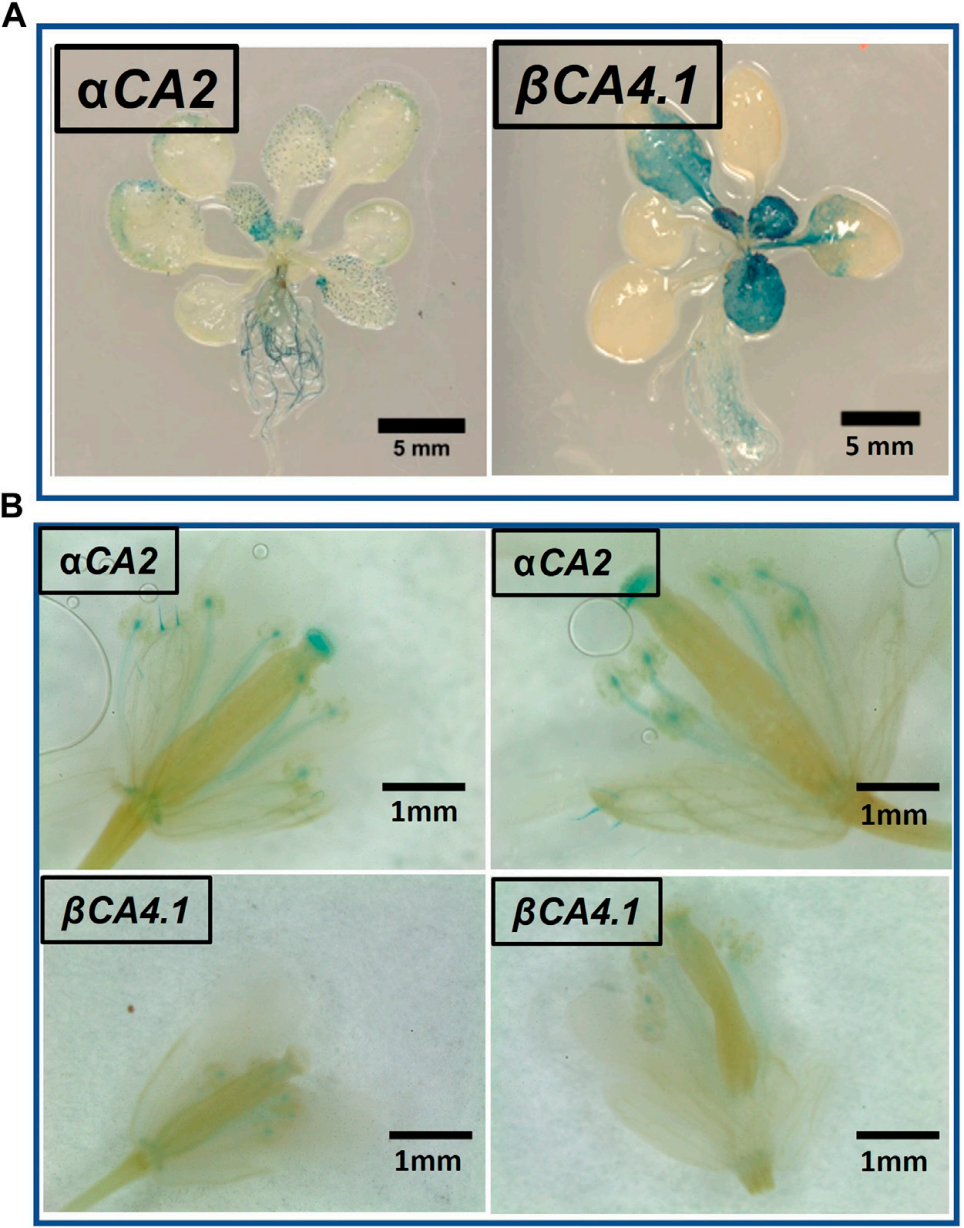
α*CA2* and *βCA4.1* expression patterns. **(A)** Vegetative tissues of three-week-old Arabidopsis plants stably transformed with either *p*α*CA2::GUS* or *pβCA4.1::GUS*. **(B)** Expression of α*CA2* and *βCA4.1* in flowers. Horizontal panels are two different flowers expressing the same gene.

To determine which plant tissues expressed *αCA2* and *βCA4.1*, promoter regions upstream of the *αCA2* and *βCA4.1* genes were used to generate promoter GUS constructs. Expression of the *pαCA2::GUS* construct showed GUS staining in roots and at the base of the young, developing leaves of the rosette (Figure 4A). In addition, there was higher expression of GUS in trichomes in plants with the *pαCA2::GUS* construct (Supplementary Figure S1). Plants containing the construct p*βCA4.1::GUS* showed strong GUS expression in both roots and leaves (Figure 4A) as has been reported earlier (DiMario et al., 2016). This result is consistent with the RNAseq data showing strong expression of βCA4 in both shoots and roots (Figure 1B). There was no significant expression of p*βCA4.1::GUS* in flowers whereas p*αCA2::GUS* showed expression in filaments and stigma (Figure 4B) in addition to the higher GUS expression in trichomes (Supplementary Figure S1).

### 3.3 Knockout plants missing αCA2 and βCA4 were obtained

**FIGURE 5:**
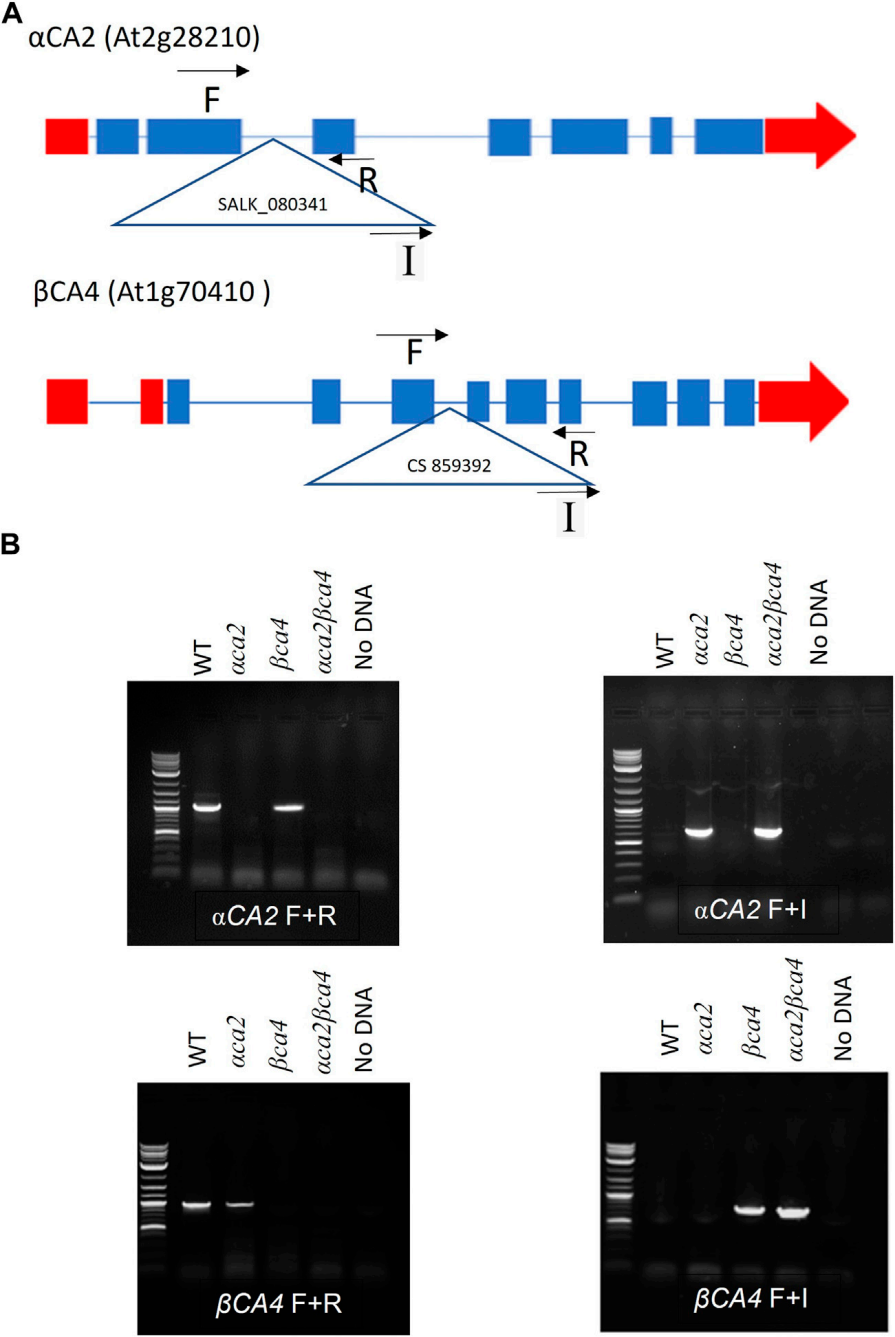
Gene maps and genomic PCRs showing *αCA2* and *βCA4* genes disruption in *βca4* and *αca2* mutants. **(A)**: Locations of the T-DNA insertions within the *αCA2* and *βCA4* genes. The *αCA2* T-DNA insertion SALK_080341 is in the second intron of the *αCA2* gene. The *βCA4* T-DNA insertion CS859392 is in the fourth intron of the *βCA4* gene. Triangles represent T-DNA insertions and black arrows represent locations of gene-specific (F and R) primers and insert (I) primers. Blue boxes and blue lines represent exons and introns, respectively. Red boxes and red arrows represent the 5′ and 3′ UTRs, respectively **(B)**; The α*CA2* and *βCA4* genes were disrupted by T-DNA insertions. Genomic PCR of WT, *αca2*, *βca4,* and *αca2βca4* plants show that the T-DNA insert SALK_080341 is present in the α*CA2* gene and the T-DNA insert CS859392 is present in the *βCA4* gene. Both inserts disrupt their corresponding genes.

**FIGURE 6:**
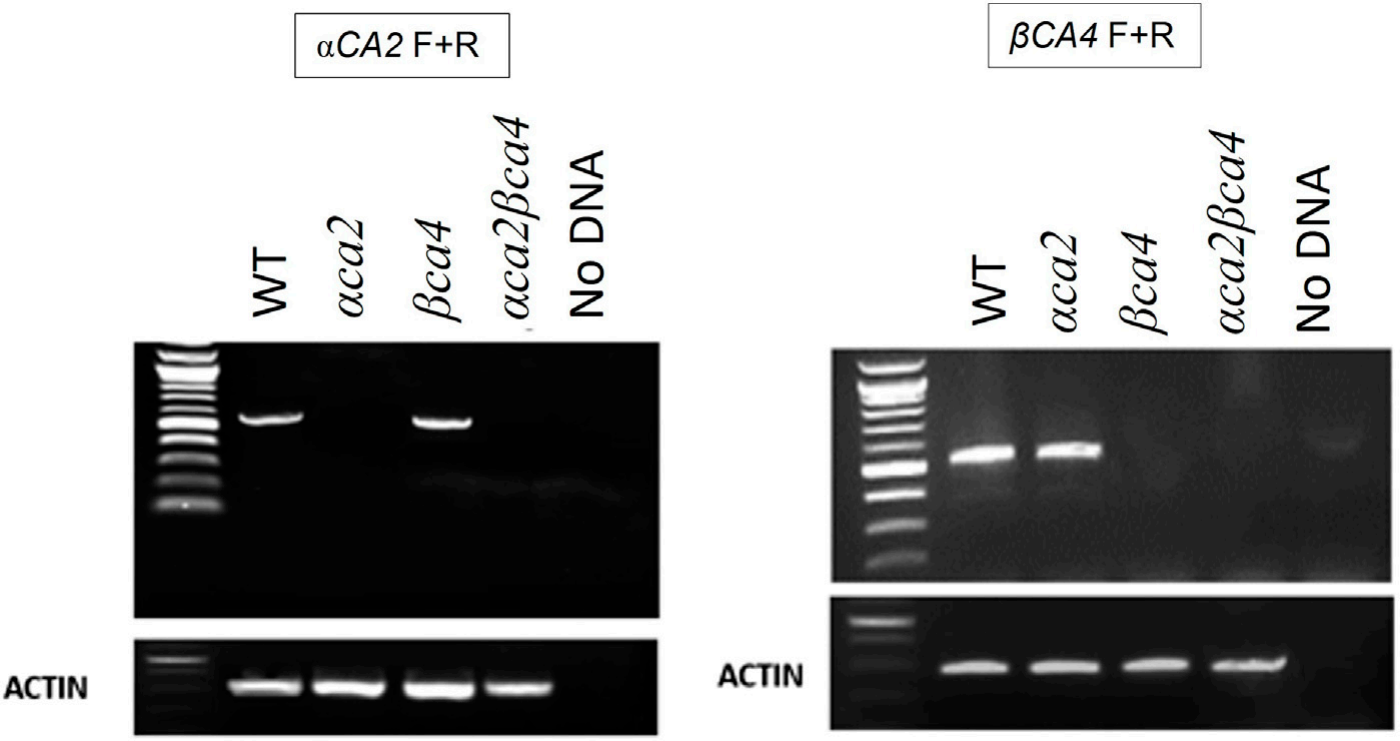
The *αca2* and *βca4* mutant lines show reduced transcription of the α*CA2* and *βCA4* genes, respectively. RT-PCR using gene-specific primers show that α*CA2* messages cannot be detected in the *αca2* and *αca2βca4* mutants and *βCA4* messages cannot be detected in the *βca4* and *αca2βca4* mutants. 3 μg of RNA was used from each sample to carry out RT-PCR.

Alleles containing T-DNA disruptions in each gene, SALK_080341 for the *αca2* line and CS859392 for the *βca4.1* line, were obtained from TAIR to determine the effect of *αCA2* and *βCA4.1* on plant growth. The SALK_080341 insert is located in the second intron of the *αCA2* gene and the CS85939 insert is located within the fourth intron of the *βCA4.1* gene (Figure 5A). Genomic PCR using either *αCA2* or *βCA4.1* gene-specific primers was used to confirm the specific T-DNA gene disruptions. In addition, a primer specific to the T-DNA insert was paired with a gene-specific primer to confirm the location of each T-DNA in its respective gene (Figures 5A, B). To confirm that these mutants are T-DNA KO lines, reverse transcription PCR (RT-PCR) was performed. The results indicated that *αCA2* and *βCA4.1* transcripts were present in the WT but absent in their respective mutant lines (Figure 6).

### 3.4 Growth of αca2βca4 plants at different CO_2_ concentrations

**FIGURE 7:**
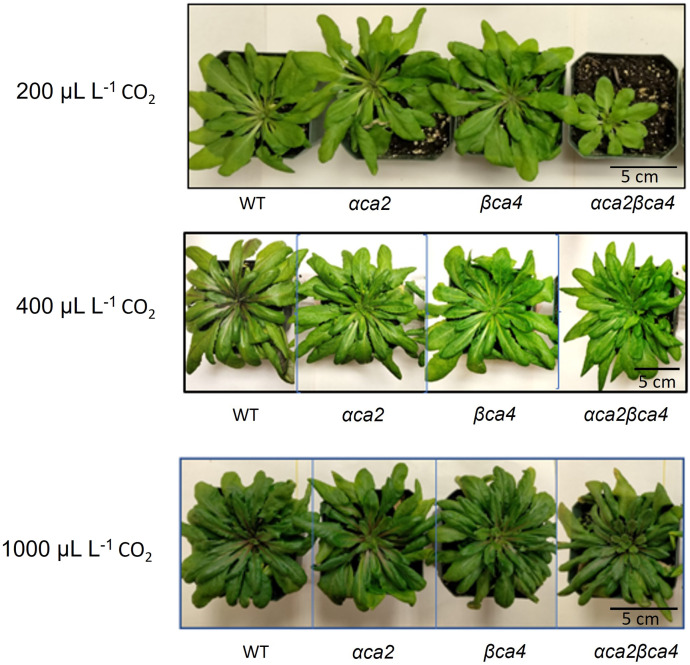
Plants disrupted in both the αCA2 and βCA4 proteins showed reduced growth at low CO_2_ (200 μL L^–1^). Images of WT, αca2, βca4, and αca2βca4 plants grown under low CO_2_ (200 μL L^–1^), ambient CO_2_ (400 μL L^–1^), or high CO_2_ (1,000 μL L^–1^) on an 8-h light/16-h dark photoperiod. Plant images were taken at the sixth week after germination.

**FIGURE 8:**
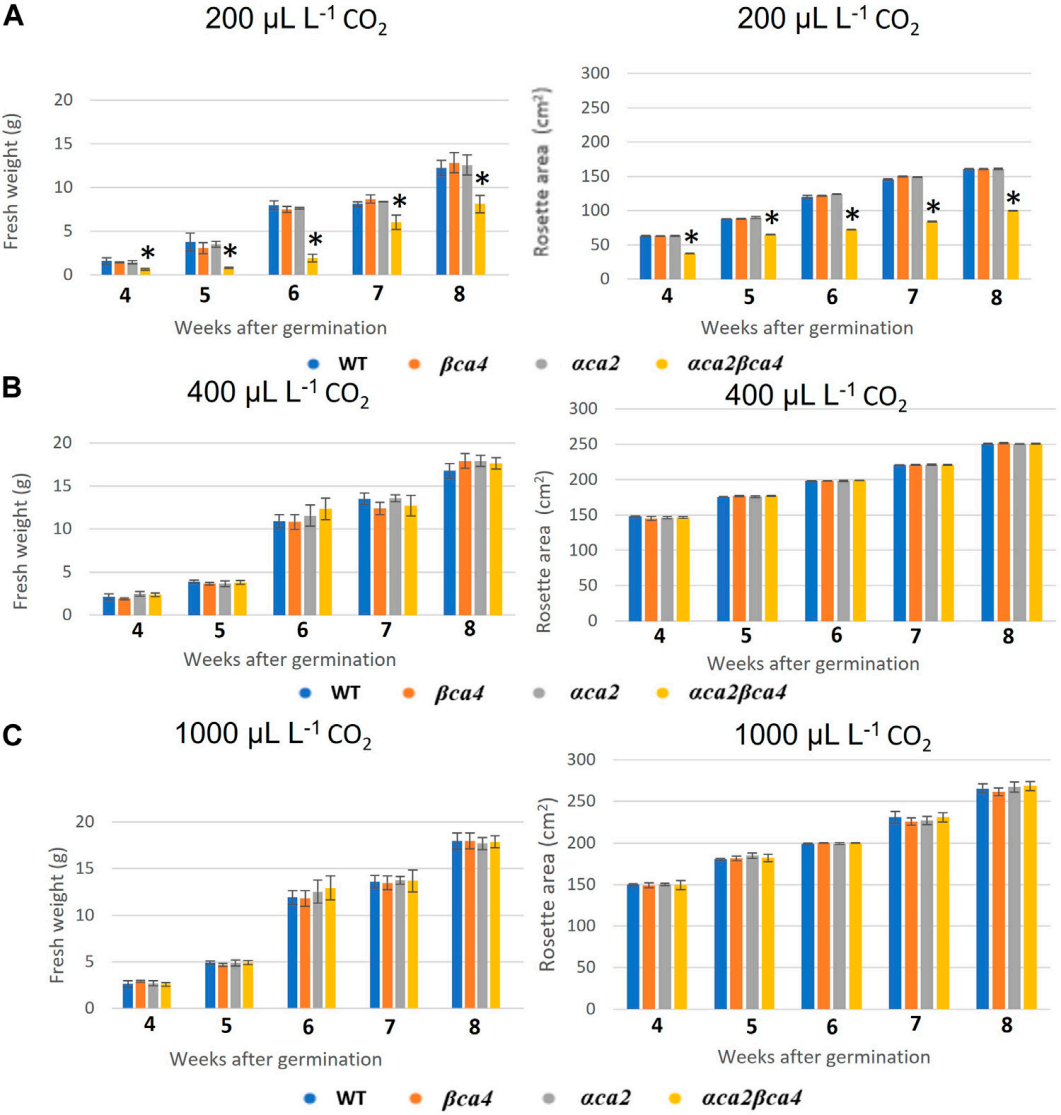
Fresh weight and rosette areas of WT, *αca2*, *βca4*, and *αca2βca4* plants grown under low CO_2_ (200 μL L^–1^), ambient CO_2_ (400 μL L^–1^), or high CO_2_ (1,000 μL L^–1^). **(A)** Average plant fresh weight and rosette area at different weeks post-germination when grown in low CO_2_ and on an 8-h light/16-h dark photoperiod. **(B)** Average plant fresh weight and rosette area at different weeks post-germination when grown in ambient CO_2_ and on an 8-h light/16-h dark photoperiod. **(C)** Average plant fresh weight and rosette area at different weeks post-germination when grown in high CO_2_ and on an 8-h light/16-h dark photoperiod. Error bars represent plus and minus one standard deviation for the means of three plants. Asterisks indicate the values that are significantly different among the four plant lines tested (*p < 0.05 by ANOVA).

Plants missing αCA2 (*αca2*), βCA4 (*βca4*), or both CAs (*αca2βca4*) were compared with WT plants when grown under different CO_2_ levels in an 8-h photoperiod with a light intensity of 120 μmol of photons m^-2^ sec^-1^. When these plants were grown under low CO_2_, (200 μL L^–1^ CO_2_) the double mutant exhibited reduced growth when compared to WT plants or the single mutants (Figure 7A and Figure 8A). When grown at low CO_2_, (200 μL L^–1^ CO_2_) the *αca2βca4* plants had a smaller rosette area and low fresh weight when compared to WT plants and the *αca2* and *βca4* lines (Figure 7A and Figure 8A). The growth of *αca2βca4* plants improved in 400 μL L^–1^ CO_2_ (ambient) or 1,000 μL L^–1^ CO_2_ (high) as it grew about the same as the WT or single mutants (Figures 7B, C; Figures 8B, C). The rosette areas and above ground fresh-weights of the WT, *αca2*, and *βca4* plants were similar at 400 μL L^–1^ CO_2_ and 1,000 μL L^–1^ CO_2_. The reduced growth was only observed in the double mutant when grown on low CO_2_.

### 3.5 αCA2 or βCA4.1 can complement the *αca2βca4* double mutants

**FIGURE 9:**
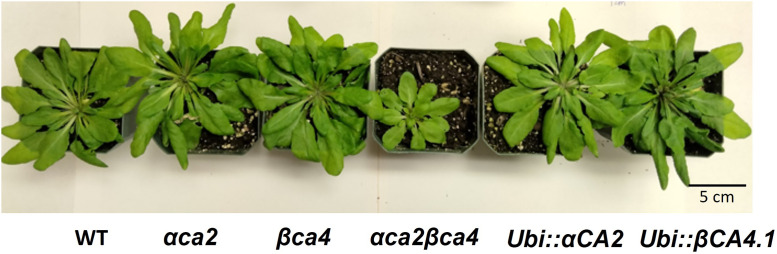
Expressing the *αCA1* and *βCA4.1* coding regions in *αca2βca4* plants restored WT growth in low CO_2_ (200 μL L^−1^ CO_2_). All plants were grown on an 8-h light/16- hour dark photoperiod with a light intensity of 120 μmol photons m^-2^ sec^-1^. The plants labeled “*ubi::αCA2*” and “*ubi::βCA4.1*” are *αca2βca4* KO plants transformed with the indicated genes driven by the ubiquitin promoter. Plant images were taken at the sixth week after germination.

To confirm whether the slow growth exhibited by the *αca2βca4* double mutant at 200 μL L^–1^ CO_2_ was due to the missing CA genes, each gene was seperately put back into the double mutant (Supplementary Figure S2). When either the WT *αCA2* or WT *βCA4.1* was transformed into the double mutant, normal growth at 200 μL L^–1^ CO_2_ was restored (Figure 9). These results agree with our earlier observations that either single mutant was indistinguishable from the WT plants under the growth conditions we employed.

### 3.6 Genotype photosynthetic response differs at low growth CO_2_

**FIGURE 10:**
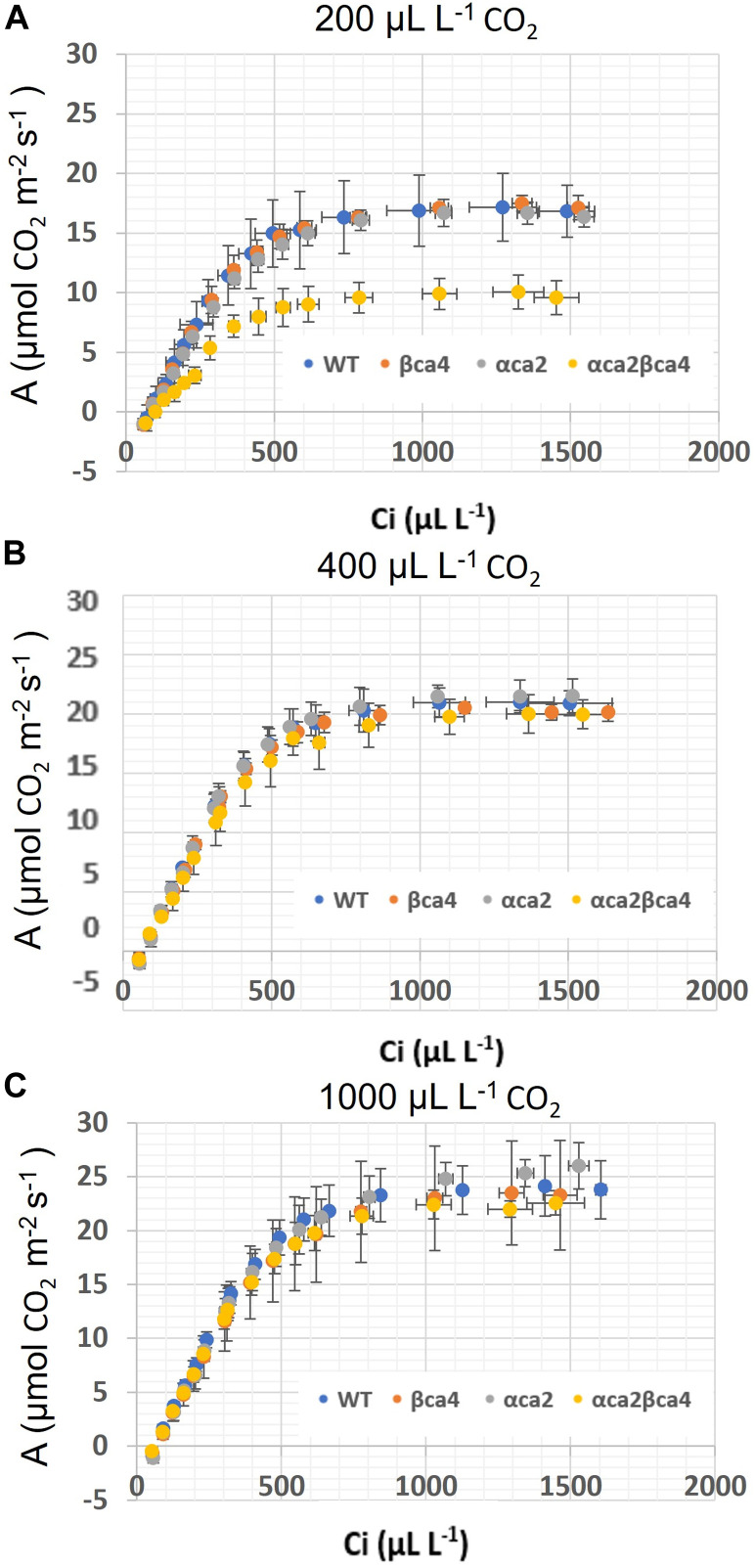
A/Ci curves for WT, *βca4*, *αca2* and, *αca2βca4* plants grown at 200, μL L^−1^ CO_2_, 400 μL L^−1^ CO_2_ and 1,000 μL L^−1^ CO_2_. **(A)** A/Ci curves for WT, *βca4*, *αca2*, and *αca2βca4* grown at 200 μL L^−1^ CO_2_. **(B)** A/Ci curves for WT, *βca4*, *αca2*, and *αca2βca4* grown at 400 μL L^−1^ CO_2_. **(C)** A/Ci curves for WT, *βca4*, *αca2*, and *αca2βca4* grown at 1,000 μL L^−1^ CO_2_. Each point is the mean of measurements of four leaves from four separate plants plus or minus standard deviation.

**FIGURE 11:**
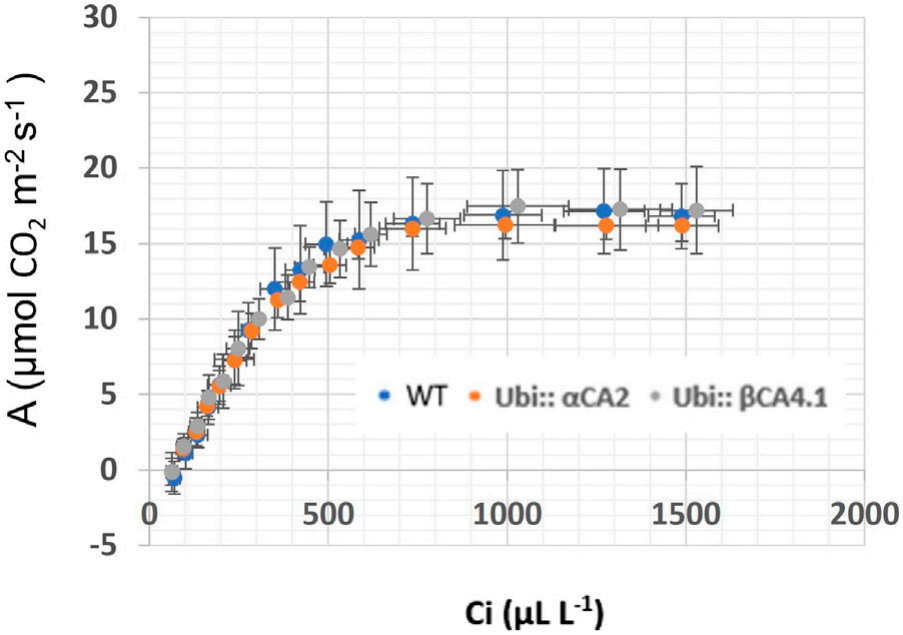
A/Ci curves for WT and *αca2βca4* complemented lines with Ubi*αCA2* and Ubi::*βCA4.*1 grown at 200 μL L^−1^ CO_2_. Each point is the mean of measurements of four leaves from four separate plants plus or minus one standard deviation. A/Ci curves were performed on the 16th youngest leaf of four independent 7-week-old plants. The CO_2_ assimilation rates of WT and *αca2βca4* complemented lines at 200 μL L^−1^ CO_2_ are not different.

The photosynthetic response of the *αca2βca4* plants was clearly different from responses of the other three genotypes when plants were grown at a CO_2_ concentration of 200 μL L^–1^ (Figure 10A). For plants growing at 400 and 1,000 μL L^–1^ the A/Ci curves were similar for all genotypes (Figures 10B, C). We further examined three characteristics of the A/Ci curves to better define the differences in photosynthetic response. At 200 μL L^–1^ the mean Acsat of the *αca2βca4* plants was 39%–42% lower than Acsat in plants from the other three genotypes (Table 1). At 400 μL L^–1^ the mean Ascat was similar among all genotypes, although the Acsat of *αca2βca4* plants increased over 100% and the Acsat of the other three genotypes increased 20%–25% from values from plants grown at 200 μL L^–1^ (Table 1). At 1,000 μL L^–1^ the Acsat of the four genotypes were again similar, however, the Acsat values again increased 6%–17.6% over the values at 400 μL L^–1^ (Table 1). For plants grown at 200 μL L^–1^, the mean initial slope of the A/Ci curves from *αca2βca4* plants was approximately 37% lower than the initial slopes from the other three genotypes (Table 2). The initial slopes from the A/Ci curves from all four genotypes were similar for plants grown at 400 and 1,000 μL L^–1^. Like Acsat, there was an increase in the initial slopes of all genotypes from 200 to 400 μL L^–1^ (57% in the *αca2βca4* and 17%–25% in the other three genotypes, Tables 1, 2). Unlike the case in Acsat, there was not an increase in the initial slopes among genotypes from 400 to 1,000 μL L^–1^ (Table 2). CO_2_ compensation points were higher in all genotypes grown at 200 μL L^–1^ CO_2_ as compared to CO_2_ compensation points in plants grown at 400 and 1,000 μL L^–1^ CO_2_. ANOVA showed the differences among growth CO_2_ concentrations was highly significant (*p* < 0.01). The mean CO_2_ compensation point for α*ca2βca4* at 200 μL L^−1^ was higher than the other three genotypes but there was not a statistical difference due to variability among replicates. The CO_2_ assimilation curves generated from the *αca2βca4* complemented lines *Ubi::αCA2* and *Ubi::βCA4.1* showed that putting back either gene results in the recovery of normal photosynthesis (Figure 11).

## 4 Discussion

Here we present evidence that αCA2 is localized to the cell wall and βCA4.1 is in the plasma membrane and show that these CAs are important in plant growth. Eliminating either αCA2 or βCA4 produces plants that are indistinguishable from WT plants. This is true whether the plants are grown at CO_2_ concentrations of 200, 400, or 1,000 μL L^–1^. However, disrupting both αCA2 and βCA4 together resulted in a plant that exhibited slow growth at 200 μL L^–1^ CO_2_ but not in plants grown at 400 μL L^–1^ CO_2_ and 1,000 μL L^–1^ CO_2_. In addition, when grown at 200 μL L^–1^ CO_2_ for 7 weeks, the double mutant had a lower capacity for photosynthesis (Figure 10 and Tables 1, 2).

The growth phenotype of *αca2βca4* plants compared to the single knockout plants supports the hypothesis that αCA2 and βCA4.1 are functioning on the same or related process. Knocking out either one of the CAs results in no observable phenotype under the growth conditions tested. However, knocking out both CAs results in plants that are unable to grow normally under 200 μL L^–1^ CO_2_ conditions (Figure 7; Figure 8). Furthermore, putting either gene back into the double mutant resulted in plants that grew normally on 200 μL L^–1^ CO_2_ (Figure 9). These results support the hypothesis that the loss of the two CAs causes the poor growth under 200 μL L^–1^ CO_2_. A similar pattern was observed in studies on CAs in the cytoplasm and in the chloroplast stroma. In the study on cytoplasmic CAs, DiMario et al. (2016) found that plants missing either βCA2 or βCA4.2 grew normally, but plants missing both proteins grew poorly under low CO_2_. In studies on the chloroplast stromal CAs, βCA1 and βCA5, both Hines et al. (2021) and Weerasooriya et al. (2022) found that both CAs had to be eliminated before serious growth defects became apparent. Therefore, it seems that it is common for CAs to have redundant or overlapping functions in plants.

**FIGURE 12:**
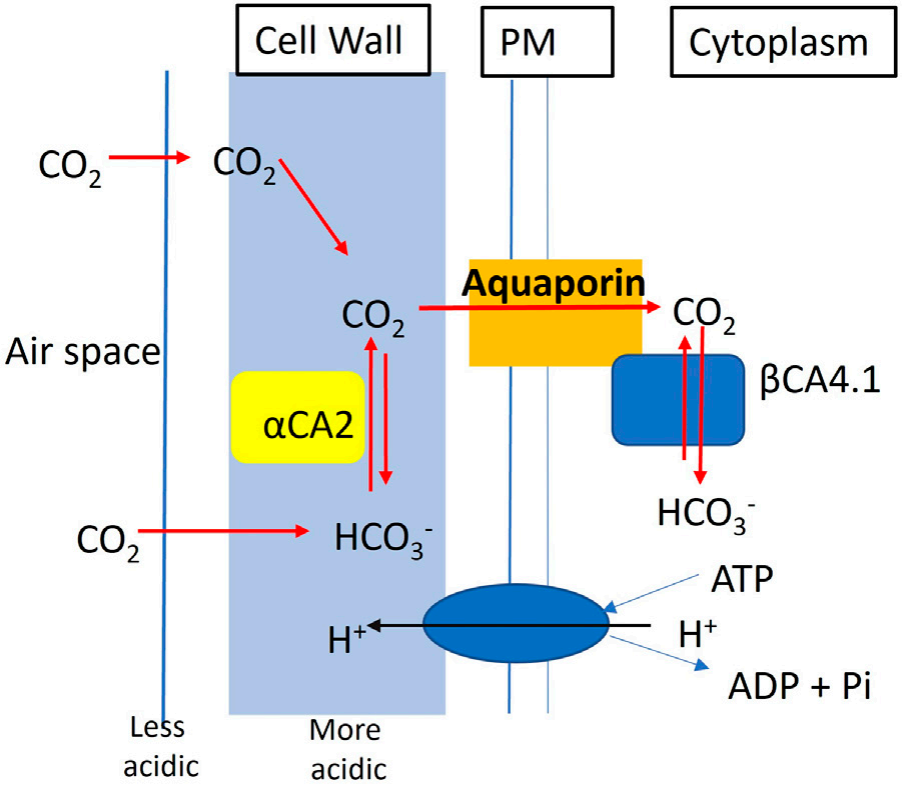
Hypothetical model showing the contribution of αCA2 and βCA4.1 in the initial CO_2_ delivery of the plant cell. αCA2 aids in delivery of CO_2_ to the aquaporin PIP2; 1, while βCA4.1 converts the CO_2_ to HCO_3_^-^ to maintain the CO_2_ gradient.

What makes this study different from the earlier studies on cytoplasmic and chloroplastic CAs, is that αCA2 and βCA4 are not localized to the same organelle. Here we present evidence that αCA2 is localized to the cell wall. This is the first report of a cell wall localized CA in plants. Clearly αCA2 would be active outside of the plasma membrane. βCA4, is found in two locations. βCA4.1 is found on the plasma membrane Fabre et al. (2007), DiMario et al. (2016), Hu et al. (2010), and Hu et al. (2015), while βCA4.2 is cytoplasmic (DiMario et al., 2016). It is thought that the active site of βCA4.1 is on the cytosolic side of the plasma membrane because it has been shown that βCA4.1 interacts with the plasma membrane aquaporin, PIP2; 1 (Wang et al., 2016). We hypothesize that αCA2 helps to replenish the CO_2_ at the cell surface for entry into the cell. Plants are often pumping H^+^ out of the cell and the cell wall near the plasma membrane is somewhat acidic. αCA2 could facilitate the production of CO_2_ from bicarbonate in the cell wall and could aid in the delivery of CO_2_ to the aquaporin, while βCA4.1 converts the CO_2_ to HCO_3_^−^ on the cytoplasmic side of the membrane. Thus, working together, αCA2 and βCA4 could be enhancing the CO_2_ gradient across the plasma membrane (Figure 12). Missing carbonic anhydrases on both sides of the membrane might disrupt the initial CO_2_ delivery to the plant cell leading to the poor growth phenotype observed when the plants are grown at 200 μL L^−1^ CO_2_.

The reason why the loss of both CAs results in poor plant growth under low CO_2_ is not clear. It is tempting to postulate that the loss of both CA directly results in reduced photosynthesis, but the data obtained from plants grown at 400 or 1,000 μL L^–1^ CO_2_ does not support that hypothesis. While it is true that CO_2_ fixation is reduced in the double mutant, this reduction is only seen when plants are grown on low CO_2_ for long periods of time. CO_2_ fixation is normal in the double mutant when it is grown on elevated CO_2_ (1,000 μL L^–1^ CO_2_) as well as on ambient air (400 μL L^–1^ CO_2_). These double-mutant plants are obviously missing both CAs, yet when grown at 400 μL L^–1^ or 1,000 μL L^–1^ CO_2_, photosynthesis is indistinguishable from that in WT and the single mutant plants even when measured at CO_2_ concentrations below 400 μL L^–1^ (Figures 10B, C, Tables 1, 2). While it is possible that the delivery of CO_2_ to Rubisco is reduced in the double mutant, the reduction in CO_2_ fixation would have to be subtle.

However, the double mutants clearly had problems when growing on reduced CO_2_. When grown at 200 µL L^–1^ CO_2_, the double mutant had a significantly lower CO_2_ saturated rate of CO_2_ fixation (Figure 10A) when compared to WT plants or the single mutants and a higher CO_2_ compensation point (Table 3). Previous work by Engineer et al., 2014 using the Arabidopsis *αca1βca4* mutant line reported that elevated CO_2_ levels increased the number of stomates in this mutant. We therefore looked at stomatal density in the *αca2βca4* double mutant but did not find a difference between the double mutant and WT plants (Supplementary Table S2). In addition, stomatal conductance was similar between the double mutant and WT plants (Supplementary Table S3).

An alternative explanation for why the double mutant is not growing well at 200 μL L^–1^ CO_2_ is that other carboxylases in the plant are adversely affected by the loss of the two CAs. The Arabidopsis genome encodes more than 20 genes with significant homology to biotin-dependent carboxylases. Most of the proteins encoded by these genes have not been studied although some are involved in important anabolic pathways such as amino acid, fatty acid, purine and pyrimidine biosynthesis. In addition, the Km (HCO_3_-) for some of these proteins is very high, often over 1 mM. Since plant tissue is at ambient CO_2_, at pH 7 or 7.5 the expected HCO_3_^−^ concentration would be in the 100–500 μM range, meaning that the plant carboxylating enzymes are experiencing suboptimal HCO_3_^−^ concentrations. So, it is possible that dropping the growth CO_2_ concentration to 200 μL L^–1^ and knocking out two of the CAs helping to deliver CO_2_ to the plant causes one or more of the other carboxylases in the plant to be adversely affected. This might lead to slower development of the plant which we see when we measure photosynthesis (Figure 10A) or fresh weight (Figure 8A). Knocking out both cytoplasmic CAs resulted in reduced amino acid biosynthesis (DiMario et al., 2016) and knocking out both chloroplastic CAs caused inhibition of fatty acid biosynthesis (Weerasooriya et al., 2022). Therefore, the reduced delivery of CO_2_ caused by knocking out αCA2 and βCA4 could lead to the reduced growth observed when the double mutant is grown on low CO_2_ for a period of many weeks.

## Data Availability

The raw data supporting the conclusions of this article will be made available by the authors, without undue reservation.
