# Supplementary material for: Carbonic anhydrases in the cell wall and plasma membrane of Arabidopsis thaliana are required for optimal plant growth on low CO2
Source: Front Mol Biosci. 2024 Feb 22;11:1267046. doi: 10.3389/fmolb.2024.1267046 (PMC10917985; doi:10.3389/fmolb.2024.1267046)
Supplement: Supplementary file 1 [file DataSheet1.PDF]

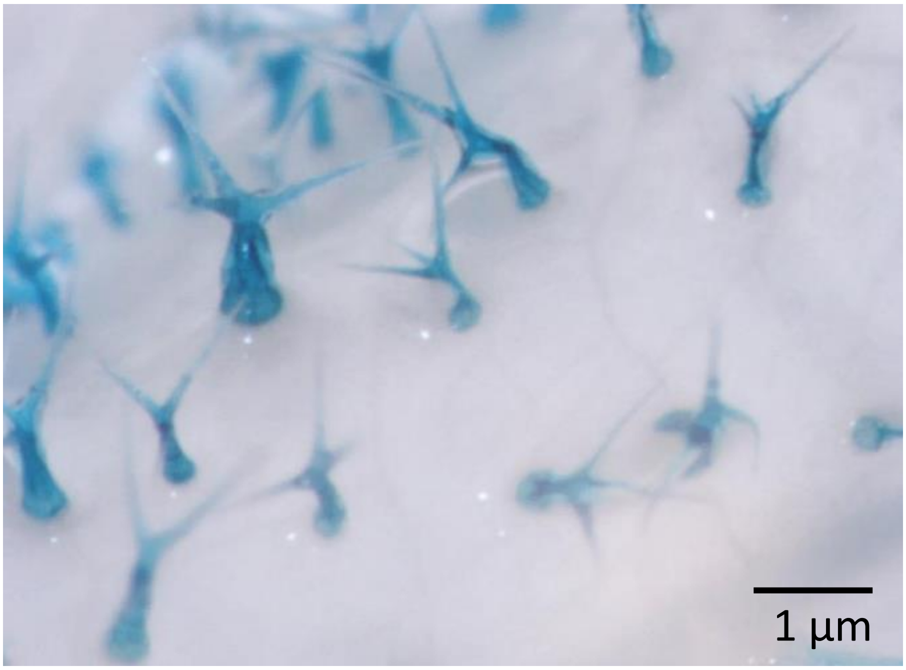

Supplemental Figure 1. GUS staining is prevalent in the trichomes of p*αCA*2::*GUS* plants. A zoomed-in image of GUS stained trichomes on a three-week-old leaf from a p*αCA2*::*GUS* plant.

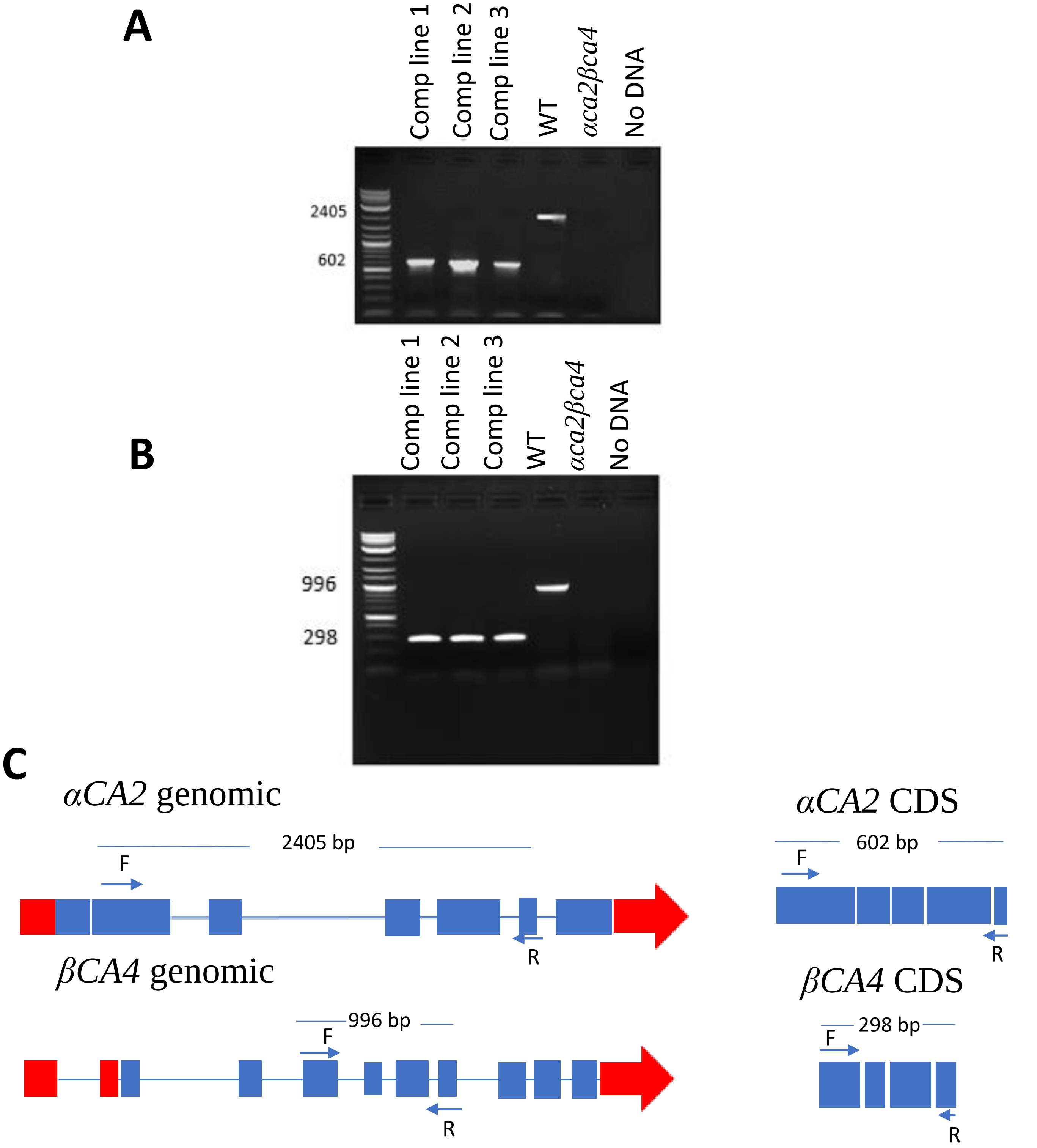

Supplemental Figure 2. Genomic PCRs confirming the presence of *αCA2* and *βCA4.1* in *αca2βca4* complemented lines A, The ubi::*αCA2* construct is present in lines 1, 2, and 3 of the *αca2βca4*-complemented lines. Native *αCA2* is present in the wild-type line while *αCA2* is missing in *αca2βca4* mutants B, The ubi::*βCA4.1* construct is present in lines 1, 2, and 3 of the *αca2βca4*-complemented lines. Native *βCA4.1* is present in the wild-type line while *βCA4.1* is missing in *αca2βca4* mutants. C. Gene maps of the *αCA2* and *βCA4* show the genomic DNA fragments and the CDS fragments amplified by the primers used to perform the above PCR.

Supplemental Table 1 . List of primers used in this study

Genomic and RT PCRs	Sequence
ACA2 F	GGTCCCAACTATTATGGTATTGC
ACA2 R	CTGTTTCGTGTTTGAGTGCAG
BCA4 F	GCGAAGATCAAAGCGTTGACG
BCA4 R	CGCCAACTCCAGAGTGTCT
Insert	ATTTTGCCGATTTCGGAAC
Genotyping complemented lines	
ACA2 F	CACCACCAAAACATTTATCTCCAATCATCACCTCTCT
ACA2 R	TAGTGATTTTGGTTTGTATAACTTTACCAC
BCA4 F	CACCATGGCTCCTGCATTCGGAAAA
BCA4 R	AGAGAAGGCAAAAGCAGGAGTGGTC

Supplemental Table 2. Stomatal density on the abaxial and adaxial sides of the WT, *βca4*, *αca2*, *αca2βca4* leaves from the plants grown at low CO_2 condition (200 μL L^{-1}). Average stomatal density values on the abaxial and adaxial sides are not significantly different from the control ($P > 0.05$ by Student's t-test). Measurements were taken from the plant lines at the seventh week after germination.

Plant line	Number of stomates abaxial side mm^{-2}	Number of stomates adaxial side mm^{-2}
WT	124 ± 4	60 ± 4
αca2	115 ± 6	65 ± 4
βca4	118 ± 8	70 ± 3
αca2βca4	120 ± 5	67 ± 5

Supplemental Table 3. Stomatal conductance values of the *WT*, *βca4*, *αca2*, *αca2βca4* plants grown at 200 µL L^{-1} CO_2. Average stomatal conductance values are not significantly different from the control ($P > 0.05$ by Student's t test). Measurements were taken from the plant lines at seventh week after germination.

Plant line	Stomatal conductance mol m^{-2} s^{-1}
WT	0.20 ± 0.042
αca2	0.16 ± 0.026
βca4	0.19 ± 0.038
αca2βca4	0.20 ± 0.041
